# Adapting to Create Innovative Virtual Falls Prevention Programs for at Risk Older Adults During a Global Pandemic

**DOI:** 10.1093/geroni/igab046.1424

**Published:** 2021-12-17

**Authors:** Maura Brennan, Rebecca Dobert

**Affiliations:** Baystate Health, Springfield, Massachusetts, United States

## Abstract

Baystate Health’s Geriatrics Workforce Enhancement Program (GWEP) postponed implementation of Group Medical Visits focused on falls reduction for older adults in Springfield, Massachusetts due to COVID-19 and quickly shifted efforts to participate in Dartmouth’s Falls Prevention Training Program. Long standing GWEP Community Based Organizations (CBOs) were consulted, and all believed that the virtual Tai Ji Quan Moving for Better Balance® (TJQMBB) program would combat social isolation and improve older adults’ comfort with technology in addition to reducing falls during the COVID-19 pandemic. Baystate’s GWEP was able to reallocate grant dollars to support the purchase of equipment for CBOs to deliver TJQMBB virtually. While many challenges continue to arise, the innovative and collaborative approach between the two GWEPs and Baystate’s CBOs leveraging Administration for Community Living falls prevention funding has led to high level engagement and rapid implementation. Dartmouth’s model capitalizes on and strengthens existing GWEP partnerships with its CBOs.

